# Milciclib-mediated CDK2 inhibition to boost radiotherapy sensitivity in colorectal cancer

**DOI:** 10.3389/fphar.2025.1557925

**Published:** 2025-03-25

**Authors:** Junjie Ma, Shanshan Wu, Xinxin Yang, Shuying Shen, Yiqian Zhu, Ruoqi Wang, Wei Xu, Yue Li, Haixin Zhu, Youyou Yan, Nengming Lin, Bo Zhang

**Affiliations:** ^1^ School of Pharmaceutical Sciences, Hangzhou First People’s Hospital, Zhejiang Chinese Medical University, Hangzhou, Zhejiang, China; ^2^ Key Laboratory of Clinical Cancer Pharmacology and Toxicology Research of Zhejiang Province, Affiliated Hangzhou First People’s Hospital, Westlake University, Hangzhou, Zhejiang, China; ^3^ Westlake Laboratory of Life Sciences and Biomedicine of Zhejiang Province, Hangzhou, Zhejiang, China

**Keywords:** Milciclib, CDK2 inhibitor, colorectal cancer, radiotherapy resistance, DNA repair

## Abstract

**Background:**

Colorectal cancer (CRC) ranks as the third most common cancer globally. Neoadjuvant radiotherapy is the standard treatment for locally advanced rectal cancer; however, primary or acquired resistance often leads to treatment failure. Identifying new targets to overcome radiotherapy resistance in CRC is crucial for improving patient outcomes.

**Methods:**

To evaluate the antitumor effects of Milciclib in CRC cells, we conducted assays measuring cell viability, cell cycle progression, and apoptosis in HCT116 and RKO cell lines following Milciclib treatment. Additionally, CRC cells were treated with a combination of Milciclib and irradiation to determine whether Milciclib could enhance their radiosensitivity. The efficacy of Milciclib was also assessed in radiation-resistant CRC cells.

**Results:**

The results of cytotoxicity and proliferation assays indicated that the IC50 values of Milciclib for human colorectal cancer cell lines HCT-116 and RKO, based on cell viability measurements, were 0.275 μM and 0.403 μM, respectively. Milciclib induced a dose-dependent reduction in the proportion of CRC cells in the G2/M phase and promoted apoptosis. When combined with irradiation, Milciclib led to a 20% increase in the proportion of cells in the G1 phase and a 10% decrease in the G2 phase, suggesting an alteration in cell cycle distribution. Additionally, Milciclib impaired DNA damage repair by inhibiting Rad51, thereby enhancing radiation sensitivity. In radiation-resistant CRC cells, the combination of Milciclib and irradiation demonstrated increased efficacy, with a sensitizer enhancement ratio (SER) above 1, indicating a potential radiosensitizing effect.

**Conclusion:**

Milciclib exhibits antitumor activity in CRC cells as a monotherapy and enhances the effectiveness of radiotherapy when used in combination. It disrupts the G2/M checkpoint and impairs DNA repair mechanisms. These findings suggest that Milciclib has the potential to be an effective therapeutic agent for CRC.

## Introduction

Colorectal cancer (CRC) is the third most common cancer worldwide and the second leading cause of cancer-related deaths. In 2020, there were approximately 1.9 million new cases of CRC globally, resulting in 935,000 deaths. By 2035, the number of CRC cases is expected to rise to 2.5 million (Sung et al., 2021; Dekker et al., 2019). Advanced CRC is characterized by a high recurrence and distant metastasis rate, with limited treatment options in chemotherapy and targeted therapies (e.g., anti-angiogenic agent bevacizumab and epidermal growth factor receptor (EGFR) inhibitor cetuximab), leading to a 5-year survival rate of only 14% (Modest et al., 2019).The currently available conventional therapies for CRC are surgical techniques, radiation, and chemotherapy treatment (Wahab et al., 2021). However, after neoadjuvant chemoradiotherapy, only 50%–60% of patients achieve partial remission, with just 15%–20% attaining a pathological complete response (pCR) (Shao et al., 2023). Some patients, particularly those with poor responses to preoperative radiotherapy, do not benefit from neoadjuvant chemoradiotherapy, leading to disease progression and delayed surgery. While it is well known that the effectiveness of radiotherapy increases with higher radiation doses, the risk of severe side effects, such as radiation-induced enteritis, limits the radiation dose in clinical practice (Van Cutsem et al., 2016). Therefore, an alternative strategy to improve clinical outcomes is to enhance tumor sensitivity to radiation. Developing effective methods to overcome radioresistance is urgently needed to achieve this goal.

CDK2, a member of the cyclin-dependent kinase family that regulates the cell cycle, plays a critical role in cell proliferation by controlling progression from the late G1 phase through the S phase (Faber et al., 2020; Zardavas et al., 2017). It is involved in various biological processes such as signal transduction, DNA damage repair, protein degradation and metabolism, and is functionally linked to the excessive proliferation and drug resistance of various cancer cells (Fagundes and Teixeira, 2021; Liu et al., 2020; Tadesse et al., 2020). Increasing evidence suggests that CDK2 contributes to radioresistance in cancer cells by enhancing DNA damage repair. CDK2 promotes homologous recombination and non-homologous end joining, facilitating the efficient repair of radiation-induced DNA damage. This enhanced repair capacity allows cancer cells to survive and proliferate despite radiotherapy, highlighting CDK2 as a potential therapeutic target for overcoming radioresistance. (Tadesse et al., 2020; Morgan and Lawrence, 2015; Deans et al., 2006; Ceccaldi et al., 2016). Additionally, CDK2 inhibitors are currently under clinical investigation for their potential to treat various cancers by inhibiting cell cycle progression (Jin et al., 2020; Satriyo et al., 2021; Wang et al., 2016). However, their clinical application faces significant challenges, including limited selectivity, toxicity, and the development of drug resistance, which necessitate further research to optimize their efficacy and safety. Milciclib is an orally bioavailable CDK inhibitor with potential antitumor activity. It effectively targets and inhibits CDK2, which may lead to cell cycle arrest and apoptosis in CDK2-expressing tumor cells (Shi et al., 2015; Lim et al., 2014). However, the effect of Milciclib on colorectal cancer (CRC), whether as a monotherapy or in combination with radiotherapy, remains unclear and requires further research.

This study aims to investigate the expression and prognostic significance of CDK2 in CRC and explore the therapeutic potential of CDK2 inhibition. Specifically, we examine the effects of Milciclib, a CDK2 inhibitor, on CRC cell cycle progression, apoptosis, and response to radiotherapy. By elucidating the underlying mechanisms, we seek to determine whether Milciclib could serve as a promising therapeutic agent for CRC, either alone or in combination with radiotherapy.

## Materials and methods

### Cell culture and drugs

Human CRC cells (Colorectal carcinoma cell lines: HCT116, RKO; Colorectal adenocarcinoma cell lines: DLD-1, HT-29) and human normal intestinal epithelial cells (NCM460) were cultured at 37°C with 5% CO_2_. The cells were maintained in 90% DMEM (HyClone, United States) or RPMI1640 medium (HyClone, United States), supplemented with 10% fetal bovine serum (HyClone, United States) and 1% penicillin-streptomycin (Life Technologies, Grand Island, NY). The stable radioresistant HCT-116RR and DLD-1RR cell lines were established from their parental HCT-116 and DLD-1 cell lines through repeated exposure to 2 Gy X-ray per day for a total of 25 fractions (typically 5 fractions per week), accumulating a total radiation dose of 50 Gy. All cell lines were purchased from the Chinese Academy of Sciences (Shanghai, China). Milciclib (HY-10424) was purchased from MedChemExpress (MCE, USA), dissolved in dimethyl sulfoxide (DMSO) (Sigma) at a concentration of 10 mM, and added to the culture medium at a final concentration not exceeding 0.1% DMSO. 3-Methyladenine (HY-19312), Z-VAD-FMK (HY-16658B), Belnacasan (HY-13205), Necrostatin-1 (HY-15760) and Ferrostatin-1 (HY-100579) were purchased from MedChemExpress (MCE, United States).

### Cell viability assay

Each of the tested cell lines was seeded into 96-well plates at a density of 3000 cells per well and incubated overnight in a complete medium. Following a 72-h drug treatment, cell viability was measured using the Cell Counting Kit-8 (CCK8) (MCE, United States) according to the protocol and assessed with a microplate reader (BioRad, United States) at an absorbance of 450 nm. The method of action of programmed cell death inhibitors: The two groups of colon cancer cells were seeded in 96-well plates at a density of 3000 cells per well. After 24 h, various programmed cell death inhibitors were added to each column (each column had 6 replicate wells), including the autophagy inhibitor 3-Methyladenine (1 μM), the apoptosis inhibitor Z-VAD-FMK (20 μM), the necroptosis inhibitor Necrostatin-1 (2 μM), the ferroptosis inhibitor Ferrostatin-1 (1 μM), and the pyroptosis inhibitor Belnacasan (1 μM), and pretreated for 6 h. Then, 400 nM Milciclib was added to each column, and the cells were treated for an additional 72 h. Cell proliferation activity was assessed using the CCK-8 assay. Assays were performed on three independent experiments.

### Colony formation assay

The effect of varying concentrations of Milciclib on tumor cells was evaluated using a colony formation assay. Briefly, 1 × 10^3^ cells were cultured in 6-well plates and treated with different concentrations of Milciclib (200, 400, 800 nM). After 72 h, the media was replaced with fresh drug-free medium and then the plate was incubated at 37°C, until visible colonies formed. The cells were then fixed with 4% paraformaldehyde (PFA, Solarbio, Beijing, China) for 15 min, followed by staining with 0.1% crystal violet at room temperature for 30 min. After washing with phosphate buffered saline (PBS, Bio-Channel, Jiangsu, China), colonies were observed under a microscope (ECLIPSE TS100, Nikon, JAPAN). Colonies containing more than 50 cells were counted using ImageJ software 1.53a version. Assays were performed on three independent experiments.

### Clonogenic survival assay

The parental HCT116 and DLD-1 cells, along with their radiation-resistant counterparts, HCT116-R and DLD-1-R, were cultured in six-well plates and subjected to a single-dose irradiation of 0, 2, 4, or 8 Gy. The initial seeding densities were adjusted according to the radiation dose, with 2000 cells for 0 Gy, 3000 cells for 2 Gy, 4000 cells for 4 Gy, and 6000 cells for 8 Gy. Cells were irradiated using a Precision X-RAD 225 machine operating at 225 kV and 13.3 mA with a 2-mm Al filter (source-to-skin distance (SSD)): 36 cm; dose rate: 1.3 Gy/min). The medium was changed 2 days after irradiation. 2 weeks post-irradiation, the plates were fixed with 4% PFA for 15 min, stained with 0.1% crystal violet for 30 min, and then washed with PBS. The linear quadratic (LQ) model (SF = exp (-aD-bD^2^)) was used to fit the survival curves. Each experiment included three duplicate wells and was performed thrice.

### Cell cycle assay

After exposure to Milciclib (with or without radiotherapy) for 24 h, the adherent cells were harvested and washed three times with PBS. It is important to note that floating dead cells in the supernatant were not collected, which may result in an underestimation of the sub-G1 population and represents a limitation of this study. The harvested cells were then fixed in 75% pre-cooled ethanol and incubated overnight at −20°C. Following three additional washes with PBS, the cells were stained with propidium iodide (PI)/RNase solutions using a commercial cell cycle detection kit (BD Biosciences) at room temperature for 15 min in the dark. The stained cells were analyzed using the BD FACS Canto II (BD Biosciences) within 1 h. Data analysis was performed using ModFit LT version 5.0 software (Verity Software House, Topsham, ME). Assays were performed on three independent experiments.

### Cell apoptosis assay

Cells were cultured in 6-well plates and treated with Milciclib, radiotherapy, or a combination of both for 72 h. Following treatment, all cells were collected and washed with PBS. They were then double-stained using the Annexin V-Phycoerythrin (PE) kit and the FITC apoptosis detection kit (BD Biosciences, Erembodegem, Belgium) for 30 min. Apoptotic cells, including both early (Annexin V+/PI−) and late apoptosis (Annexin V+/PI+), were detected by flow cytometry (BD Biosciences) within 1 h. The total apoptotic rate was calculated as the sum of early and late apoptotic cells. Data analysis was performed using FlowJo version 10.8.1 software (FlowJo, Oregon). Assays were conducted in three independent experiments.

### Western blot analysis

Cells were seeded in 6-well plates at a density of 2 × 10^5^ cells per well and lysed using Radio Immunoprecipitation Assay Lysis buffer (RIPA buffer, Beyotime, China) supplemented with 1 x protease inhibitor (Solarbio, China) and phosphatase inhibitor (Solarbio, China). The proteins were denatured by boiling in a 1.5 × SDS loading buffer. Equal amounts of proteins were separated by SDS-PAGE and transferred to NC membranes. The membranes were blocked at room temperature with 5% non-fat milk and then incubated overnight at 4°C with primary antibodies. After washing with Tris-buffered saline containing Tween 20 (TBST), the membranes were incubated with secondary antibodies at room temperature for 1 h. Following another wash, an ECL reagent was applied, and the membranes were analyzed using the Bio-Rad Molecular Imager ChemiDoc XRS + system (BioRad, United States). We have normalized the Western blot results by comparing the grayscale values of the target protein to those of the housekeeping protein in the same sample to ensure consistency. Regarding the concentration of Milciclib, we determined it based on the IC50 values in 2 cell lines, selecting three concentrations on either side of the IC50 (200, 400, and 800 nM) for treatment. To investigate the time-dependent effects of Milciclib on colon cancer cells, we first conducted cell cycle and apoptosis experiments on RKO cells, choosing time points of 24, 48, and 72 h (sub 1D,E). The results showed that Milciclib significantly inhibited the cell cycle in RKO cells at 24 h, so we selected 24 h as the time point for detecting cell cycle-related proteins. In the apoptosis experiment, colon cancer cells showed significant apoptosis at 72 h, so we selected 72 h as the time point for detecting apoptosis-related proteins.

The primary antibodies against CDK2 (#2546T), phosphor-CDK2 (THr160, #2561S), phospho-H2AX (S139, #9718T), CyclinE (#20808T), β-Actin (#4970T), PARP (#9532S), Cleaved PARP (#5625S), Bcl-2 (#3498S), Bax (#5023T), Rad51 (#8875T), Rad50 (#3427T) and GAPDH (#2118T) were purchased from Cell Signaling Technology (Danvers, MA), The secondary antibodies against, anti-rabbit (A0208) and anti-mouse (A0216) were purchased from Beyotime (Shanghai, China).

### Statistical analysis

The data were analyzed and visualized using GraphPad Prism 8.0, Modfit 5, FlowJo, and SPSS 26.0 software. All experiments were performed in triplicate, and the results are presented as mean ± SD. Statistical analysis was conducted using the t-test, with a p-value of less than 0.05 considered statistically significant. The significance levels are indicated as follows: *p < 0.05, **p < 0.01, ***p < 0.001, ****p < 0.0001.

## Results

### Phosphorylated CDK2 expression is upregulated in colorectal cancer cells

**FIGURE 1:**
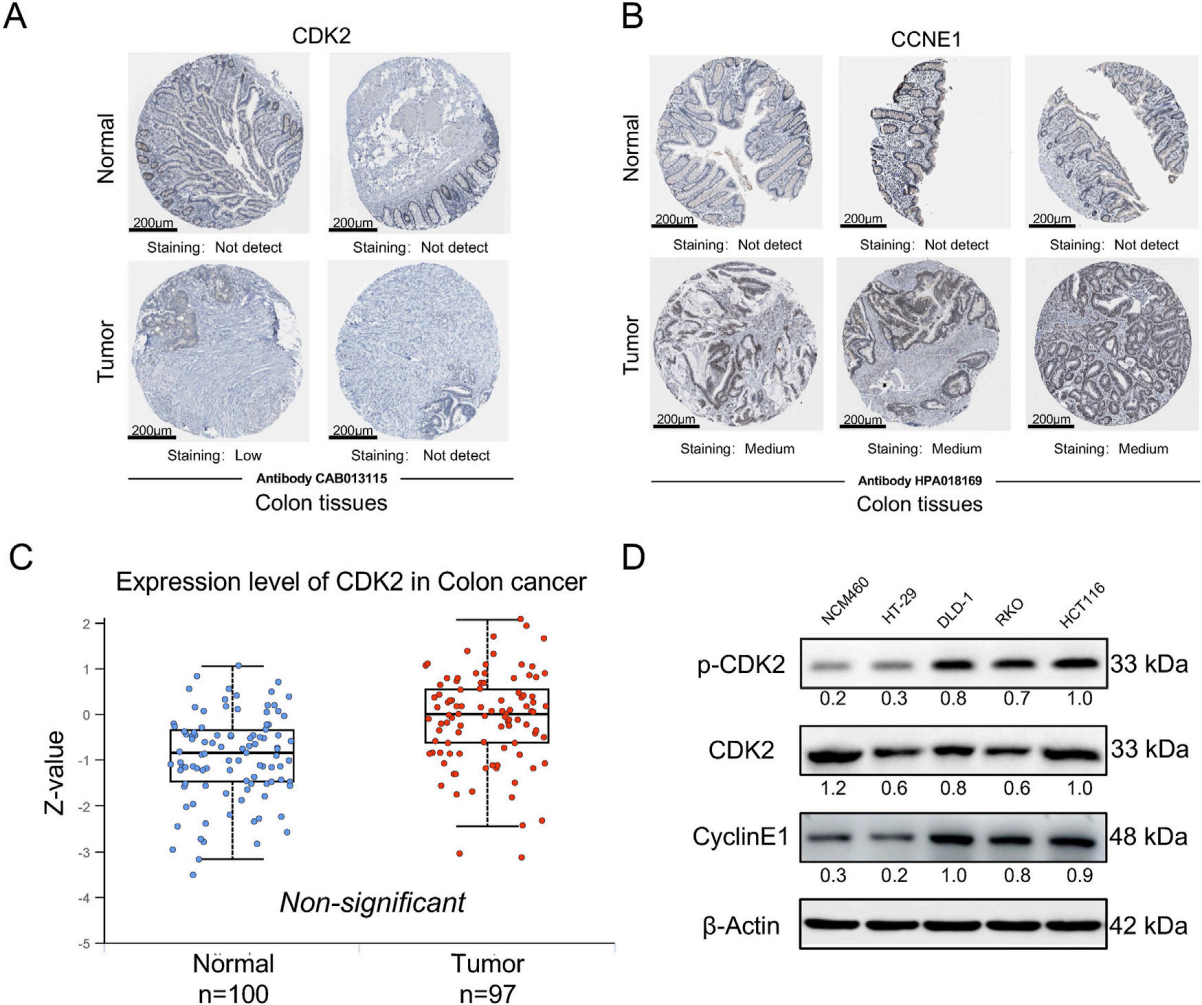
p-CDK2 and CCNE1 expression in CRCs tissues at protein levels **(A, B)**. Immunohistochemical tissue chip images of CDK2 and CCNE1 proteins obtained from the HPA database. **(C)** The expression of total CDK2 protein in normal and cancer tissues was obtained from the HPA website CPTAC database **(D)**. Western blot analysis of p-CDK2 and CyclinE1 expression in NCM460, HCT116, RKO, HT-29, and DLD-1 cells.

We initially assessed the expression of CDK2 and CCNE1 (the gene encoding the G1/S phase-specific cell cycle protein Cyclin E1) in human cancers using public databases. The results presented here are based, in whole or in part, on data generated by the UALCAN platform (https://ualcan.path.uab.edu). In colorectal cancer (CRC), both CDK2 and CCNE1 were significantly upregulated (sub 1A,B). Compared to normal tissues, the expression levels of these genes were markedly higher in cancerous tissues (sub 1C). Additionally, we examined the protein expression levels of CDK2 and CCNE1 in normal and cancerous intestinal tissues using the HPA database (https://www.proteinatlas.org). The immunohistochemistry results showed no difference in total CDK2 protein levels between normal and tumor tissues, while CCNE1 protein expression exhibited significant differences in the tumor group (Figures 1A, B). Subsequently, we re-evaluated the expression differences of total CDK2 protein levels between normal and tumor groups using the CPTAC database on the HPA website. The results showed that there was no significant difference in total CDK2 protein expression between the two groups (Figure 1C). This discrepancy suggests that RNA and protein expression may be regulated by different mechanisms, particularly post-transcriptional modifications. Although CDK2 RNA expression was elevated in cancerous tissues, its protein levels may be influenced by post-translational modifications, such as phosphorylation. Given that CDK2 function is contingent on its phosphorylation status, we hypothesized that alterations in CDK2 phosphorylation might underlie these observations. To investigate this further, we analyzed the expression of phosphorylated CDK2 and total CDK2 in normal intestinal epithelial cells and colorectal cancer cells. The results demonstrated a significant upregulation of phosphorylated CDK2 in colorectal cancer cells (Figure 1D).

### The CDK2 inhibitor Milciclib induces cell cycle arrest by inhibiting activated CDK2 in colorectal cancer

**FIGURE 2:**
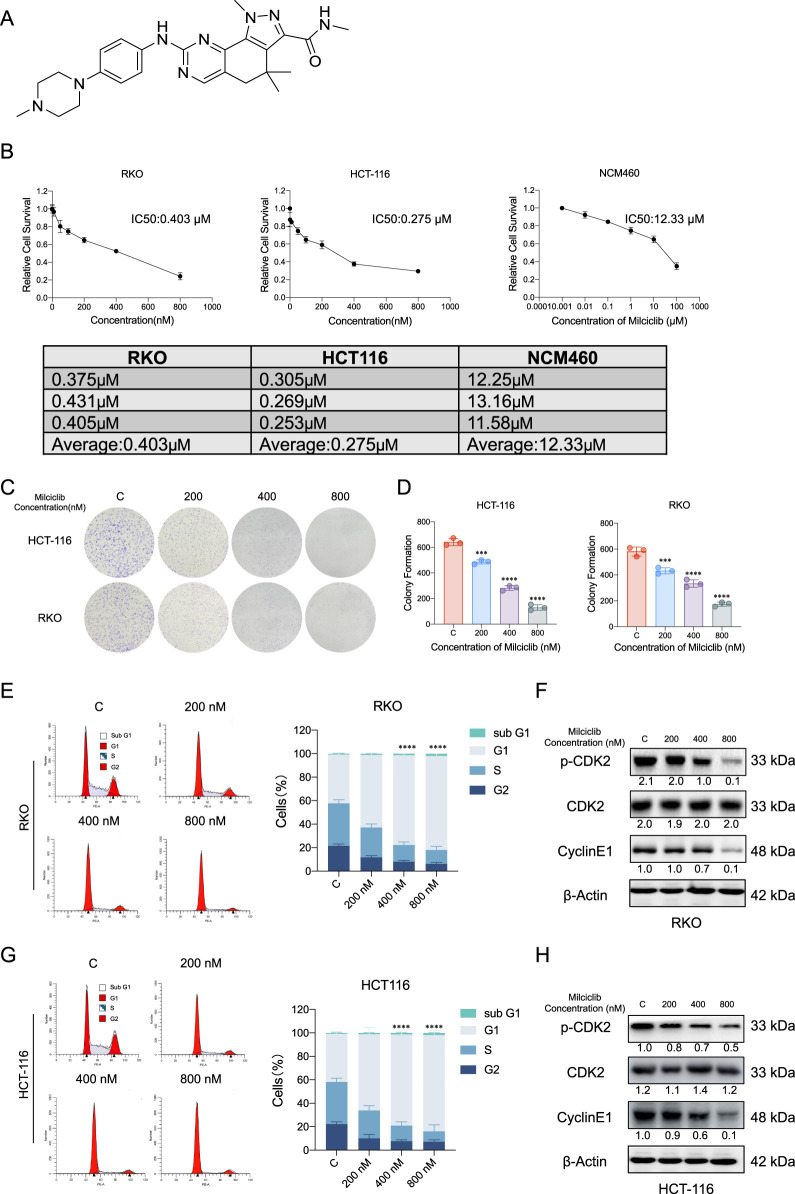
Milciclib blocks G1/S phase of the cell cycle in a dose-dependent manner **(A)**. Chemical structure of Milciclib **(B)**. The IC50 of Milciclib in RKO, HCT116 and NCM460 cells **(C, D)**. Colony formation assay of CRC cells treated with varying concentrations (0 nM, 200 nM, 400 nM, and 800 nM) of Milciclib **(E, G)**. Flow cytometry analysis indicates Milciclib-induced G1 phase arrest in CRC cells **(F, H)**. Western blot analysis shows the inhibition of cell cycle-related proteins in CRC cells mediated by Milciclib. The statistical significance shown in the figure is compared to the control group. *P < 0.05, **P < 0.01, ***P < 0.001, ****p < 0.0001.

To evaluate the anti-tumor effects of Milciclib in CRC cells, we assessed cell viability at various concentrations of Milciclib (Figure 2A). The results showed that the IC50 values of Milciclib in two CRC cell lines were 0.275 μM for HCT116 and 0.403 μM for RKO, which were lower than those in NCM460 cells (Figure 2B). This difference may be related to the elevated expression of p-CDK2 and CCNE1 in CRC cells. A dose-escalation colony formation assay conducted with Milciclib in HCT116 and RKO cells revealed a decrease in the number of cell colonies as the dose of Milciclib increased (Figures 2C, D). Since CDK2 is a critical kinase in the G1/S phase of the cell cycle, we further explored the mechanism of action of Milciclib in CRC cells by treating HCT116 and RKO cells with different concentrations of the drug (200, 400, and 800 nM). After 24 h of treatment, cells were collected for flow cytometry to analyze cell cycle distribution. The results indicated that Milciclib induced G1 phase arrest in a concentration-dependent manner, as evidenced by an increasing percentage of cells in the G1 phase. This was accompanied by a corresponding decrease in the percentage of cells in the S and G2/M phases. Additionally, after 24 h of treatment with 800 nM Milciclib, the percentage of cells in the sub-G1 phase remained low, with RKO cells at 1.9% and HCT116 cells at 1.85%. (Figures 2E–G). Additionally, protein samples were collected for Western blot analysis to detect the expression levels of cell cycle-related markers, and the results were consistent with those from the flow cytometry analysis (Figures 2F–H).

### Milciclib induces apoptosis in CRC cells

**FIGURE 3:**
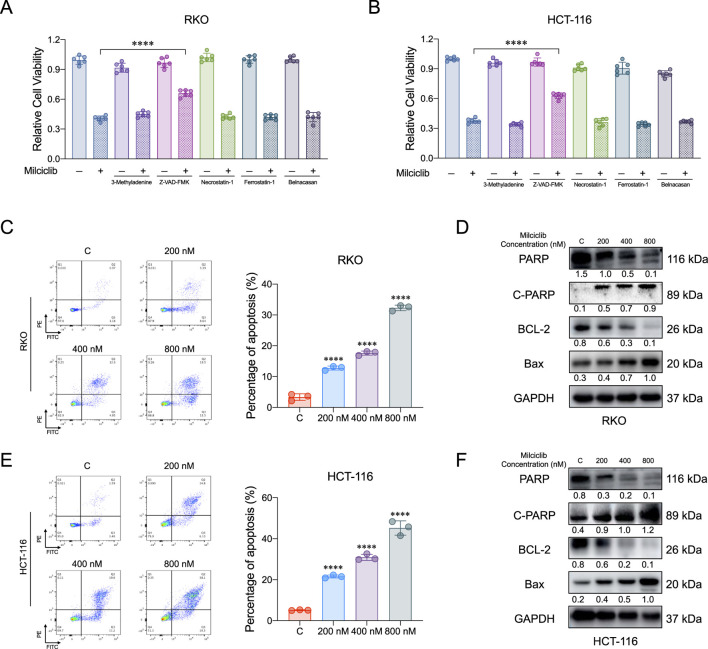
Milciclib induce apoptosis in a dose-dependent manner **(A, B)**. Cells were pretreated with various cell death inhibitors for 6 h, followed by co-incubation with Milciclib for 72 h, and cell proliferation was subsequently assessed using the CCK-8 assay **(C, E)**. Milciclib induced apoptosis in CRC cells in a concentration-dependent manner (The statistical significance shown in the figure is compared to the control group) **(D, F)**. Changes in the expression of apoptosis-related proteins were induced by Milciclib. *P < 0.05, **P < 0.01, ***P < 0.001, ****p < 0.0001.

To further investigate the mechanism by which Milciclib affects CRC cells, we pretreated HCT116 and RKO cells with various inhibitors for 6 h: autophagy inhibitor 3-Methyladenine (1 μM), apoptosis inhibitor Z-VAD-FMK (20 μM), necroptosis inhibitor Necrostatin-1 (2 μM), ferroptosis inhibitor Ferrostatin-1 (1 μM), and pyroptosis inhibitor Belnacasan (1 μM). After pretreatment, the cells were incubated with 400 nM Milciclib for 72 h, and their proliferative activity was measured using the CCK-8 assay. The results showed that the apoptosis inhibitor partially reversed the proliferation inhibition of HCT116 and RKO cells induced by 400 nM Milciclib, while the autophagy, necroptosis, ferroptosis, and pyroptosis inhibitors had no such effect (Figures 3A,B). To confirm that Milciclib induces apoptosis in CRC cells, we performed apoptosis assays on HCT116 and RKO cells. Annexin V staining demonstrated that the proportion of apoptotic cells significantly increased in a dose-dependent manner after 72 h of Milciclib treatment at different concentrations (Figures 3C–E). This finding was further supported by dose-dependent alterations in apoptosis-related proteins. As the Milciclib concentration increased, there was a marked upregulation of the pro-apoptotic protein Bax and cleaved PARP, accompanied by a downregulation of both PARP and Bcl-2 proteins. (Figures 3D–F).

### The combination of Milciclib and radiotherapy may enhance the anti-tumor effect

**FIGURE 4:**
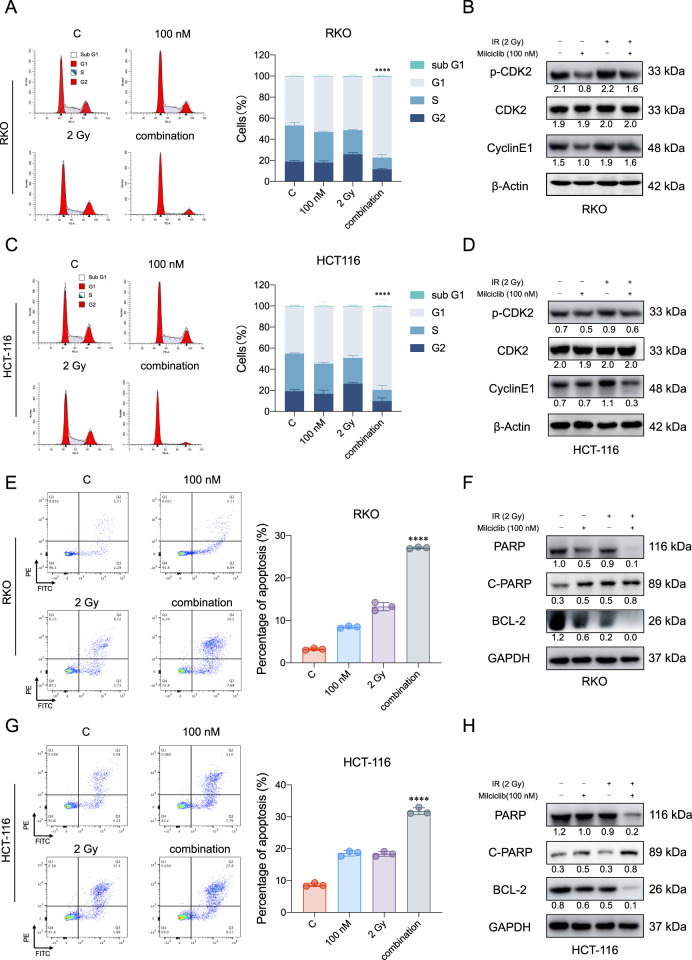
Combination therapy of Milciclib and radiotherapy enhances apoptosis in CRCs **(A, C)**. The combination of Milciclib and radiotherapy induces G1/S phase cell cycle arrest **(B, D)**. Changes in cell cycle-related proteins caused by the combination of Milciclib and radiotherapy **(E, G)**. The combination of Milciclib and radiotherapy increases the apoptosis rate in CRC tumor cells **(F, H)**. The combination of Milciclib and radiotherapy alters the levels of apoptosis-related proteins. The statistical significance shown in the figure represents comparisons between the combined radiotherapy and drug treatment group *versus* the radiotherapy-only and drug-only treatment groups, respectively. *P < 0.05, **P < 0.01, ***P < 0.001, ****p < 0.0001.

Currently, the first-line clinical treatment for CRC involves a combination of chemotherapy and radiotherapy. To evaluate the effect of combining Milciclib with radiotherapy, HCT116, and RKO cells were cultured in six-well plates and Milciclib was added 24 h before irradiation. 24 h post-irradiation, cells were collected for flow cytometry and Western blot analysis. Flow cytometry results showed that Milciclib induced G1 arrest in CRC cells, with a significant reduction in the proportion of cells in the G2 phase (Figures 4A–C). Western blot analysis further confirmed these results, revealing a downregulation of key G1/S phase proteins, including CDK2, p-CDK2, and cyclin E1 in the combination treatment group (Figures 4B–D). The disruption of the cell cycle also triggered apoptosis, and the combination of Milciclib with irradiation exhibited an accumulative effect in CRC cells (Figures 4E, G). Western blot results were consistent with flow cytometry findings, showing a downregulation of Bcl2 and PARP expression, alongside an upregulation of cleaved PARP expression (Figures 4F, H).

### Milciclib partially reversed the radioresistance of cells by inhibiting CDK2

**FIGURE 5:**
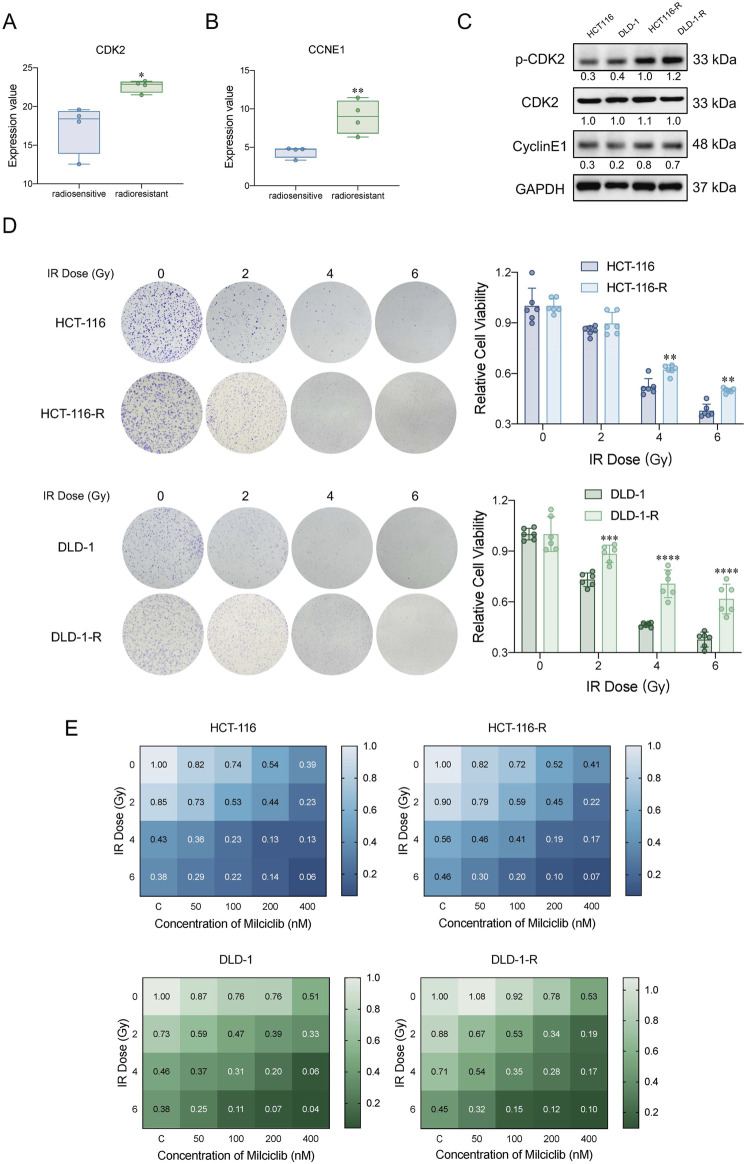
Milciclib mitigated cellular radioresistance by partially inhibiting CDK2 activity **(A, B)**. Expression differences of CDK2 and CCNE1 between radiotherapy-sensitive and -resistant patients in the GSE186940 dataset analyzed via RNASeq (n = 4). (The statistical significance shown in the figure represents the comparison between the radiotherapy-resistant group and the radiotherapy-sensitive group) **(C)**. Western blot analysis of CDK2 and CyclinE1 expression in HCT116-R and DLD-1-R cells **(D)**. Clonogenic survival assay of HCT116-R and DLD-1-R cells under different irradiation doses. (The statistical significance shown in the figure represents the comparison between the radiotherapy-resistant group and the parental group) **(E)**. Proliferative activity of CRC cells and radiotherapy-resistant cells treated with Milciclib in combination with radiotherapy, assessed using the CCK-8 assay. The Sensitization Enhancement Ratio (SER) is calculated by comparing the effect of a combination treatment (e.g., a drug plus radiation) to the effect of the treatment alone (e.g., radiation alone) on a specific biological endpoint, such as cell viability. In other words, it compares how much more effective the combination treatment is compared to the treatment used alone. SER >1 = Synergistic effect (combining Milciclib with radiotherapy enhances the effectiveness of radiotherapy).*p < 0.05, **p < 0.01, ***p < 0.001, ****p < 0.0001.

We analyzed whole-genome sequencing data from the GEO gene expression database (https://www.ncbi.nlm.nih.gov/geo/), comparing 4 radiotherapy-tolerant patients to 4 radiotherapy-sensitive patients (Zhou et al., 2023). The analysis revealed that CDK2 and CCNE1 were more highly expressed in radiotherapy-tolerant patients than in those sensitive to radiotherapy (Figures 5A,B). To validate these findings, we established radiotherapy-resistant strains of HCT116 and DLD-1 cells (named HCT116-R and DLD-1-R). Using a colony formation assay, we confirmed their radioresistance (Figure 5D). We further examined the protein levels of CDK2 and CCNE1 in the radioresistant cells, which aligned with our initial analysis from the GEO database (Figure 5C). To assess Milciclib’s effect on these radioresistant cells, HCT116, DLD-1, and their radioresistant strains were treated with varying concentrations of Milciclib 24 h before irradiation. After 72 h of gradient irradiation, cell proliferation activity was measured using the CCK-8 assay. In the HCT116 parental cell line, the Sensitization Enhancement Ratio (SER) for 100 nM Milciclib combined with 2Gy radiation compared to 2Gy radiation alone was 1.68. In the DLD-1 parental cell line, the SER for the same combination was 1.88. In the HCT116 radiation-resistant cell line, the SER for 100 nM Milciclib combined with 2Gy radiation compared to 2Gy radiation alone was 1.67, while in the DLD-1 radiation-resistant cell line, the SER was 1.78. These results suggest that Milciclib can enhance the sensitivity to radiotherapy, thereby improving the effectiveness of radiotherapy (Figure 5E).

### Milciclib induces apoptosis in radiation-resistant colorectal cancer cells by inhibiting RAD51

**FIGURE 6:**
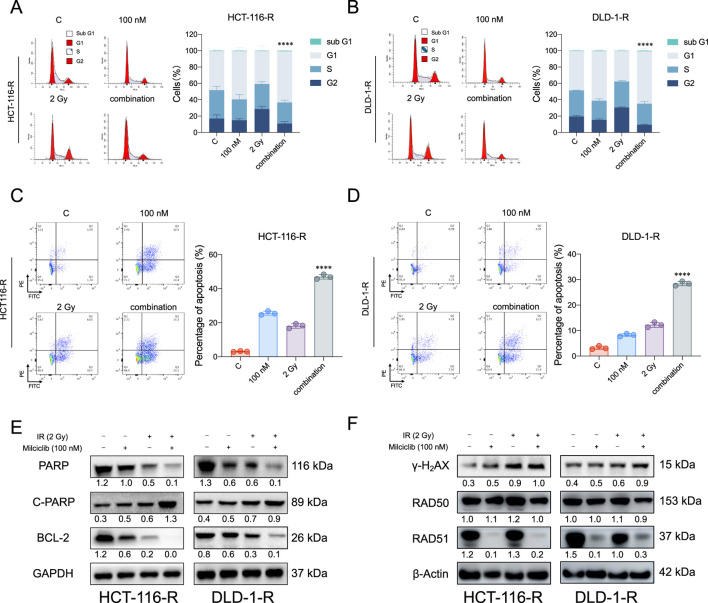
Milciclib promotes apoptosis in radiation-resistant colorectal cancer cells by suppressing RAD51 activity **(A, B)**. The combination of Milciclib and radiotherapy reduces the proportion of G2/M phase cells in radiotherapy-resistant cells **(C, D)**. The combination of Milciclib and radiotherapy increases the apoptosis rate in CRC radiotherapy-resistant cells **(E)**. The combination of Milciclib and radiotherapy alters the levels of apoptosis-related proteins **(F)**. The combination of Milciclib and radiotherapy inhibits the expression of proteins involved in damage repair. The statistical significance shown in the figure represents comparisons between the combined radiotherapy and drug treatment group *versus* the radiotherapy-only and drug-only treatment groups, respectively. *p < 0.05, **p < 0.01, ***p < 0.001, ****p < 0.0001.

To assess the impact of Milciclib on radiation-resistant colorectal cancer (CRC) cells, HCT116-R and DLD-1-R cells were seeded in six-well plates and treated with Milciclib 24 h before irradiation. After 24 h of irradiation, cells were collected for flow cytometry and Western blot analysis. Flow cytometry results revealed that the combination of Milciclib and radiation significantly altered the cell cycle distribution. While irradiation alone caused a prominent G2/M phase arrest, the addition of Milciclib further enhanced this arrest, with a notable reduction in the proportion of cells in the G2/M phase compared to irradiation alone (Figures 6A, B). This suggests that Milciclib may synergize with radiation by impairing the cells’ ability to repair DNA damage, thereby amplifying the cytotoxic effects.

Furthermore, this disruption in cell cycle progression was closely associated with an increase in apoptosis. Both flow cytometry (Figures 6C, D) and Western blot analysis confirmed that the combination treatment induced a significantly higher apoptotic rate than either Milciclib or irradiation alone. Specifically, Western blotting showed a downregulation of anti-apoptotic proteins such as Bcl-2 and PARP, while cleaved PARP levels were upregulated, indicating a marked increase in apoptosis following the combination treatment (Figure 6E).

Given the critical role of DNA damage in radiation-induced cell death, we further investigated the effect of Milciclib on the DNA damage repair pathways in resistant cells. Western blot analysis demonstrated a significant upregulation of γ-H2AX (H2A histone family member X, a well-established marker of DNA double-strand breaks), in cells treated with the combination of Milciclib and irradiation. In contrast, RAD51, a key protein involved in homologous recombination repair of DNA breaks, was notably downregulated, suggesting that Milciclib may impair the DNA repair machinery, thereby enhancing the DNA damage caused by irradiation (Figure 6F). These results highlight the cooperative effects of Milciclib and irradiation in disrupting the cell cycle, promoting apoptosis, and inhibiting DNA repair pathways in radiation-resistant CRC cells.

## Discussion

Colorectal cancer (CRC) is one of the leading causes of cancer-related mortality worldwide. Despite advancements in treatment modalities, the clinical prognosis of CRC patients remains poor. Radiotherapy, as one of the crucial treatment strategies, is often challenged by radioresistance. Tumor cells counteract radiation-induced DNA damage during radiotherapy by activating DNA repair mechanisms and cell cycle checkpoints, thereby enhancing their radioresistance (Morgan and Lawrence, 2015). Therefore, overcoming radioresistance and improving tumor sensitivity to radiation have become key issues in improving the prognosis of CRC patients.

Currently, chemotherapy combined with radiotherapy (chemoradiotherapy) is one of the most effective strategies for treating various human cancers, particularly CRC (Glynne-Jones et al., 2006; Wilson et al., 2006; Gerard et al., 2006). The combination of 5-fluorouracil (5-FU) and radiotherapy has become the standard treatment regimen in CRC (Sauer et al., 2012). Studies have shown that the synergistic effect of 5-FU and radiation has been successfully applied in the clinical treatment of several cancers, including rectal cancer, pancreatic cancer, and cholangiocarcinoma (Ojima et al., 2006). 5-FU enhances the efficacy of radiotherapy by affecting multiple biological processes. For example, 5-FU increases radiosensitivity by inducing the degradation of SIRT7, thereby reducing the proportion of cells in the S phase (Kiran et al., 2015; Tang et al., 2017). In this study, we explored the potential of Milciclib, a CDK2 inhibitor, to enhance CRC cell sensitivity to radiotherapy. Our results demonstrated that Milciclib significantly inhibited CRC cell growth when used alone, with its mechanism involving the reduction of the G2/M phase cell proportion and induction of dose-dependent apoptosis. Importantly, when combined with radiotherapy, Milciclib significantly increased the radiosensitivity of both standard and radioresistant CRC cells, as evidenced by a sensitization enhancement ratio (SER) greater than 1. This suggests that, compared to existing chemoradiotherapy strategies, Milciclib not only enhances the radiotherapeutic effect but also reduces the side effects of radiotherapy by decreasing the required radiation dose. In contrast to conventional chemotherapy drugs such as 5-FU, which typically require higher sensitizing concentrations (around 10 μM) (Tang et al., 2017), our study shows that Milciclib can effectively enhance radiotherapy efficacy at concentrations as low as 0.1 μM. This marked difference highlights the superior potential of Milciclib in enhancing radiotherapy sensitivity.

Moreover, our study also reveals that Milciclib can effectively overcome radiotherapy resistance, a topic that has not been fully explored in current research. Radiotherapy resistance is a critical factor contributing to poor clinical outcomes and tumor recurrence in CRC patients (Yao et al., 2022). Only 15%–20% of patients with tumor volume reduction after radiotherapy exhibit complete responses, and their 5-year survival rate is less than 65% (Siegel et al., 2022). Milciclib, by inhibiting CDK2, significantly reverses radiotherapy resistance, reduces the DNA repair capacity of cancer cells, and enhances radiotherapy efficacy. Previous studies have indicated that CDK2 plays a key role in both homologous recombination (HR) and non-homologous end joining (NHEJ) DNA repair pathways, and the expression of Rad51 is an important marker of HR repair (Deans et al., 2006; Ceccaldi et al., 2016). We found that Milciclib could suppress Rad51 expression, reduce the DNA repair capability of radioresistant cells and significantly enhance their sensitivity to radiation. This is consistent with previous research findings.

In conclusion, our study demonstrates that Milciclib, by targeting and inhibiting CDK2, significantly enhances CRC cell sensitivity to radiotherapy and overcomes radiotherapy resistance by suppressing the G2/M checkpoint and DNA repair mechanisms. Compared to conventional chemotherapy drugs like 5-FU, Milciclib shows a stronger radiosensitizing effect at lower concentrations. Our findings provide important experimental evidence for the application of Milciclib as a radiosensitizer in CRC treatment and offer a novel therapeutic strategy to overcome radiotherapy resistance in clinical settings.

## Data Availability

The raw data supporting the conclusions of this article will be made available by the authors, without undue reservation.
