# Supplementary figures and images for: Milciclib-mediated CDK2 inhibition to boost radiotherapy sensitivity in colorectal cancer
Source: Front Pharmacol. 2025 Mar 25;16:1557925. doi: 10.3389/fphar.2025.1557925 (PMC11975868; doi:10.3389/fphar.2025.1557925)

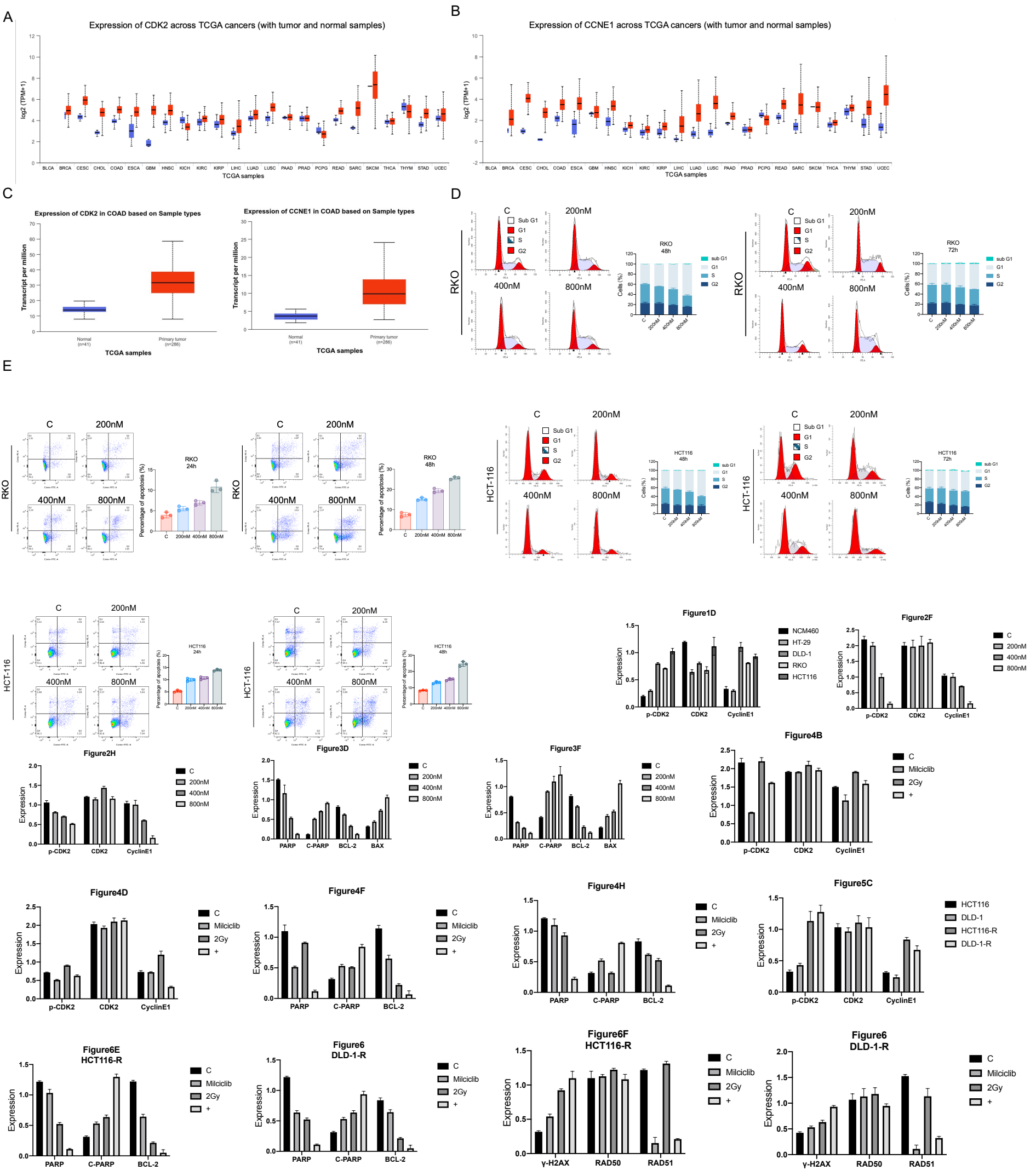

Supplement: Supplementary file 1 [file DataSheet1.pdf]
